# Analyzing Distributed Vibrating Sensing Technologies in Optical Meshes

**DOI:** 10.3390/mi13010085

**Published:** 2022-01-05

**Authors:** Saifur Rahman, Farman Ali, Fazal Muhammad, Muhammad Irfan, Adam Glowacz, Mohammed Shahed Akond, Ammar Armghan, Salim Nasar Faraj Mursal, Amjad Ali, Fahad Salem Alkahtani

**Affiliations:** 1 Electrical Engineering Department, College of Engineering, Najran University Saudi Arabia, Najran 61441, Saudi Arabia; msakond@nu.edu.sa (M.S.A.); snmursal@nu.edu.sa (S.N.F.M.); fsalkhtani@nu.edu.sa (F.S.A.); 2 Department of Electrical Engineering, Qurtuba University of Science and IT, D. I. Khan 29050, Pakistan; 3 Department of Electrical Engineering, University of Engineering Technology, Mardan 23200, Pakistan; 4 Department of Automatic Control and Robotics, Faculty of Electrical Engineering, Automatics, Computer Science and Biomedical Engineering, AGH University of Science and Technology, al. A. Mickiewicza 30, 30-059 Krakow, Poland; adglow@agh.edu.pl; 5 Department of Electrical Engineering, College of Engineering, Jouf University, Sakaka 72388, Saudi Arabia; 6 Department of Electrical Engineering, Jalozai Campus, University of Engineering and Technology, Peshawar 24240, Pakistan; amjadalikhalil@gmail.com or

**Keywords:** frequency division multiplexing, phase optical time/frequency domain reflectometry, optical meshes, distribution vibration sensing

## Abstract

Hundreds of kilometers of optical fibers are installed for optical meshes (OMs) to transmit data over long distances. The visualization of these deployed optical fibers is a highlighted issue because the conventional procedure can only measure the optical losses. Thus, this paper presents distributed vibration sensing (DVS) estimation mechanisms to visualize the optical fiber behavior installed for OMs which is not possible by conventional measurements. The proposed technique will detect the power of light inside the optical fiber, as well as different physical parameters such as the phase of transmitted light inside the thread, the frequency of vibration, and optical losses. The applicability of optical frequency domain reflectometry (OFDR) and optical time-domain reflectometry (OTDR) DVS techniques are validated theoretically for various state detection procedures in optical fibers. The simulation model is investigated in terms of elapsed time, the spectrum of a light signal, frequency, and the impact of many external physical accidents with optical fibers.

## 1. Introduction

### 1.1. Motivation

**Figure 1:**
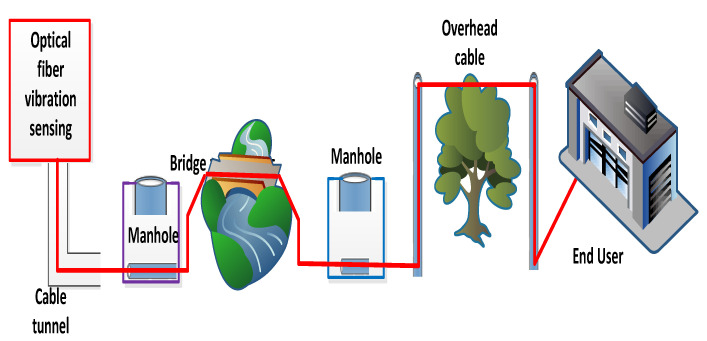
The installation of optical fiber communication system and external disturbance which creates losses inside the optical fiber.

Several techniques to identify the dispersion and scattering issues inside optical fibers have been employed for a long-distance communication system, including their locations [1,2]. Out of these studied techniques, many researchers have addressed optical frequency domain reflectometry (OFDR) and optical time-domain reflectometry (OTDR) measuring procedures, which measure optical fiber losses with their positions [3]. Enhancing the estimating sensitivity, distance and resolutions are the key objectives of these mechanisms. If an abnormality or failure occurs inside the optical fiber, these techniques are used to identify the losses and location with the help of optical power [4,5]. The calculations of temperature and static strain distribution in the field of optical fibers have been investigated for many years [6]. Moreover, for measuring the dynamic strain of an optical fiber, a vibration procedure is employed on the optical fiber in terms of time variation estimations of the dispersed light waveform. The function of the fiber sensor is to recognize the fault in the attached fiber. For this purpose, the vibration measurement technique is considered a fruitful method which replicates the ability of hammering test [7]. The aim of this work is to design an optical fiber sensor network for all of society. The improvement and features of fiber vibration sensors are studied for optical fiber communication. The installed optical fiber for communication has physical contact in some places with the external environment. Therefore, the vibration is experienced by optical fiber continuously. The framework of an employed optical fiber for a communication system and its relationship with the external environment is explained in Figure 1. This declares that fiber cables applied for optical communications are disturbed in view of physical vibrations. These vibrations have the potential to identify the fault and its location [8]. Based on these properties of vibration, this paper investigates the development of distribution vibration sensing (DVS) technologies using OTDR and OFDR procedures for evaluating the fault inside the optical fiber. When measuring the vibration of an optical fiber, it is difficult to detect different states of vibrations for determining failure in optical fiber communication. Thus, it is necessary to measure the vibration and state of the fiber quantitatively and to estimate the complex amplitude of light dispersion inside the optical fiber. Taking these points in this model, we have investigated the detection of fiber vibration and its meaningful outcomes like the hammer test. The vibration measuring schemes based on frequency multiplexed OTDR are studied, including averaging the waveform to improve vibration measurements. Finally, the estimations of vibration in terms of OFDR and OTDR are compared to present which parameter gives efficient results.

### 1.2. Major Contribution

Conventional techniques for identifying the troubles are used only for estimations of the optical losses. The mechanism of DVS measurements, aiming to visualize the behavior of the optical fiber and implemented for an optical communication system is studied in this paper. The significant contributions of this work are given as follows.

1. DVS measurement technologies are proposed for visualizing the state of the optical fiber;
2. The significant drawbacks of OFDR, like the low repetition rate of the probe light and short measurement range, are addressed in this paper;
3. The single-sideband modulation through a microwave signal generator is applied to manage the repetition rate, and relative distance estimations are used to improve distance measurements;
4. The analytical analysis of the proposed model is investigated in terms of short-time Fourier transform (STFT), optical frequency, laser phase noise, backscattered signal light and fiber range.

### 1.3. Comparative Distance Computation Using OFDR

**Figure 2:**
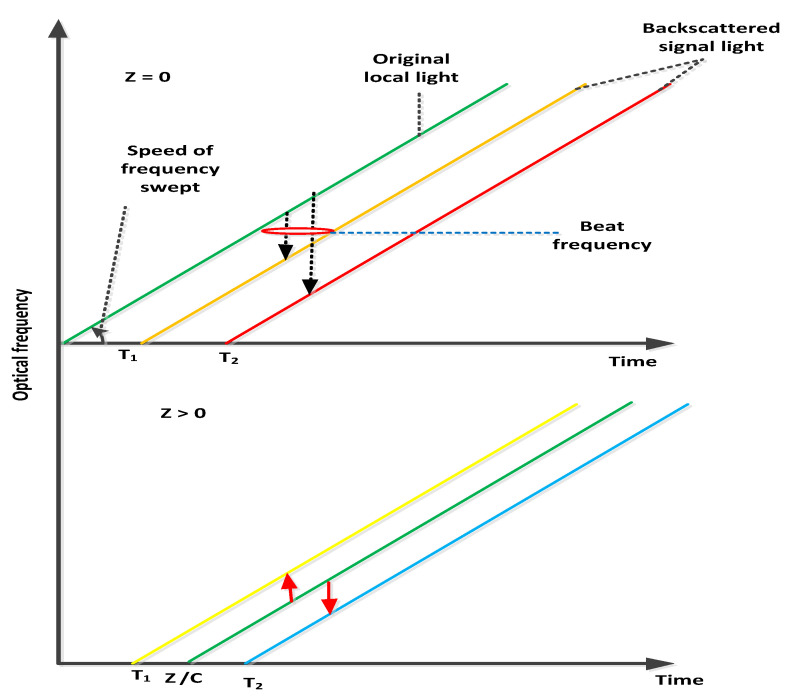
Relative distance estimations.

**Figure 3:**
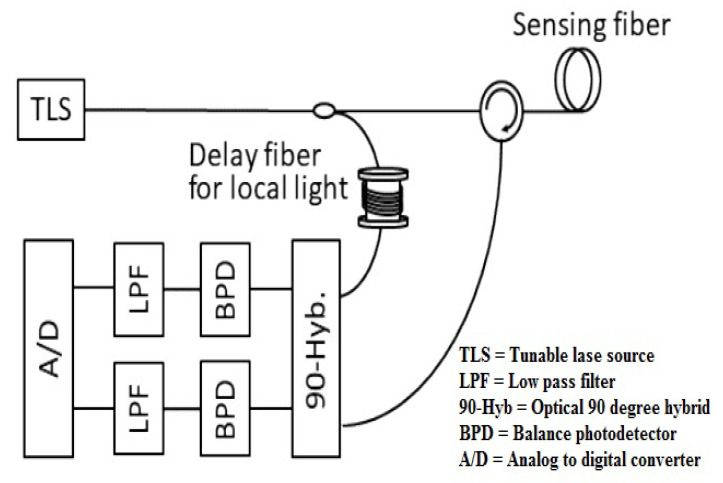
Comparative distance estimations by the OFDR framework.

The source light of frequency swept is utilized by OFDR and calculates the beat wave among the backscattered and local lights. This presents the delayed replica of local light, the path length of which shows the OFDR reference distance. The comparative delay $\tau_{r}$ among backscattered and local lights give the beat frequency $\vartheta_{beat}$ [9,10] and is documented as

(1) $$\vartheta_{beat}=\zeta \tau_{r}=\zeta \frac{2(\mu -\mu_{l})}{v}$$

where ζ, μ, $\mu_{l}$ and *v* denote the frequency-swept speed, backscattered light distance, local light path length and light speed in optical fibers, respectively. The conceptual background of the beat frequency is depicted in Figure 2 with and without local light *Z* path length. At Z=0, the beat frequency of the traditional OFDR system is single sided and proportional to the fiber under test (FUT) length. On the other side, the beat frequency is proportional to the relative distance for the Z>=0 condition among the distance of the backscattered and half distance of the local lights. The need for a high bandwidth receiver is decreased using this terminology for identifying a distance location. In addition, the beat frequency allocated by the relative distance is double sided and consists of positive and negative frequencies, which corresponds to shorter and longer distances. Equation (1) performs the conversion from the relative to the absolute domains [11,12]. The calculation model is presented in Figure 3, which shows that the relative distance estimation incorporates a delayed optical fiber for local light. It is also depicted in Figure 3 that a 90 degree hybrid optical is selected for discriminating the positive and negative beat frequencies. The purpose of installed low pass filter in the model described in Figure 3 is to minimize the aliasing induced in the transmitted signals. The temporal waveform of the electric field E(t) and frequency swept [13,14] are written as

(2) $$E\left(t\right)=exp\left\{i[2\pi \left(f_{0}+\frac{1}{2}\zeta t\right)+\phi \left(t\right)]\right\}.$$

Here, $f_{0}$ declares the initial optical frequency, and the laser phase noise is denoted by ϕ. The postponed fiber $E_{1}\left(t\right)$ is propagated by local light [15,16,17] and defined as

(3) $$E_{l}\left(t\right)=E\left(t-\tau_{l}\right),E_{s}\left(t\right)=\int r\left(t\right)E\left(t-\tau \right)d\tau ,$$

where $E_{s}\left(t\right)$ defines the backscattered signal light.

(4) $$\tau_{l}=\frac{\mu_{l}}{v}$$

(5) $$\tau =\frac{2\mu }{v}$$

In Equations (4) and (5), $\tau_{1}$ and τ represent the delay generated by the local delay fiber and delay round trip, respectively. In addition, an optical 90-degree hybrid resolves the beat signals bs(t) among $E_{1}\left(t\right)$ and $E_{s}\left(t\right)$ into in-phase and quadrature components [18,19,20], which are written as

(6) I(t)=Re[bs(t)]

(7) Q(t)=Im[bs(t)]

(8) $$bs\left(t\right)=E_{l}\left(t\right)\cdot E_{s}\left(t\right)$$

(9) $$E_{l}\left(t\right)\cdot E_{s}\left(t\right)=\int d\tau exp\left[i-2\pi (f_{0}+\zeta t\left(\tau -\tau_{l}\right))\right].$$

The in-phase and quadrature elements are denoted by I(t) and Q(t), respectively. The beat signals discussed in Equation (9) consist of the optical frequency response and are calculated by centering local delay. Furthermore, the Fourier transfer is used for measuring the reflection coefficient distribution r(τ) in the local fiber length. In correlation with traditional OFDR framework, the phase noise difference π(t−τ)−π (*t*$\tau_{1}$) is calculated by absolute distance [21]. In the proposed system, two process are involved for measuring phase noise difference: (1) the relative distance of the local delay fiber is used for attaining the phase noise difference; and (2) the achieved phase noise difference is then suppressed at the centering location of the local delay fiber. The discussed principles are confirmed by the experimental setup as mentioned in Figure 3, where a 5.2 km stable fiber is used for the sensing fiber and a 65 m length fiber is selected for vibration purposes. In addition, a limit of a 30 Hz DC bandwidth is kept for the vibration waveform. In order to generate the frequency swept light, external single side band (SSB) modulation [22,23] is applied in the OFDR model. As a result, the short estimation and low repetition rate impairments are minimized. The laser (<1 KHz linewidth, 1550 nm), signal generator, IQ modulator, band pass filter and frequency multiplier parameters are installed for the frequency swept light generator. Similarly, optical 90-degree hybrid, low pass filter, photodetector and analog to digital converter elements are applied for backscattered light at the receiving side. The vibrations, including the fiber stretcher, are synchronized in the proposed model and accumulate the frequency modulation on the probe light coherently. The accumulated frequency modulation behaves like a beat frequency modulation that interrogates the estimation of various positions. The second achievement of this model is that a shorter frequency sweep time (510 μs, 2.2 GHz bandwidth) is adopted for suppressing the beat frequency modulation. The high repetition rate with 380 Hz is the third fruitful outcome of this proposed DVS rating.

**Figure 4:**
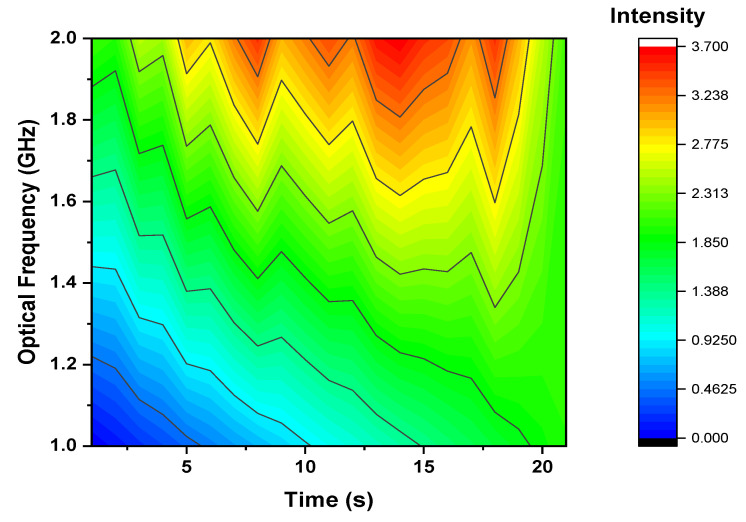
Optical frequency measurement in terms of time at 5000 m. fiber length.

The framework of the OFDR is customized in this paper to examine the path and estimate the vibrations. The presented setup includes two properties. The first is single side modulation, which creates frequency swept light from a laser with a narrow linewidth. As a result, the Nyquist frequency of DVS estimations and range are developed. The second is that a long delay fiber is applied for local light, through which the measurement range and path positions are analyzed. The vibration estimation analyses are declared in Figure 4 at a distance of 5000 m.

### 1.4. Organization and Notaion of Paper

The rest of the paper is organized as follows. Section 2 gives the discussion of the proposed model, and Section 3 provides the conclusion of the proposed DVS technologies.

## 2. Discussion

**Figure 5:**
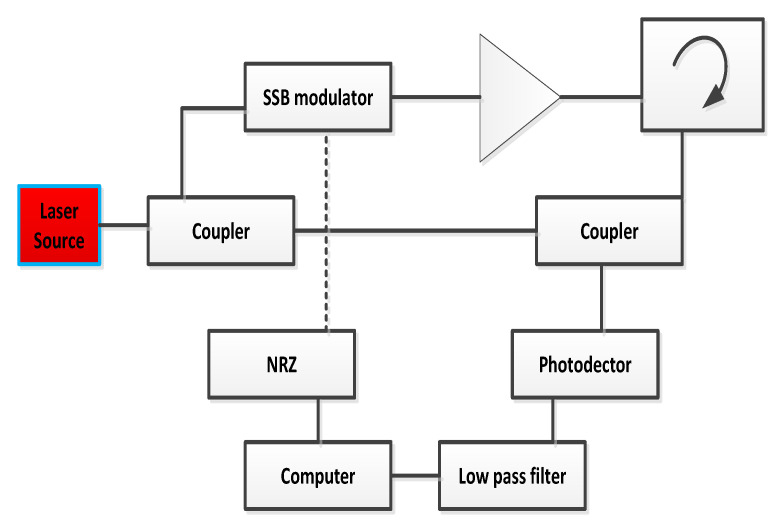
OTDR-coherent FDM-based vibration sensing classifications.

**Figure 6:**
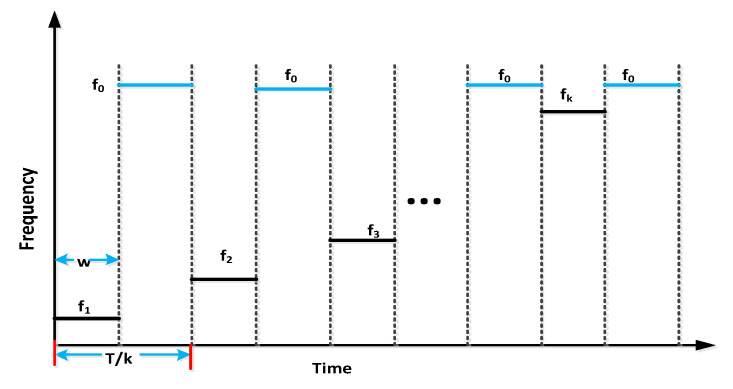
Analysis of a sequence based on frequency coding.

**Figure 7:**
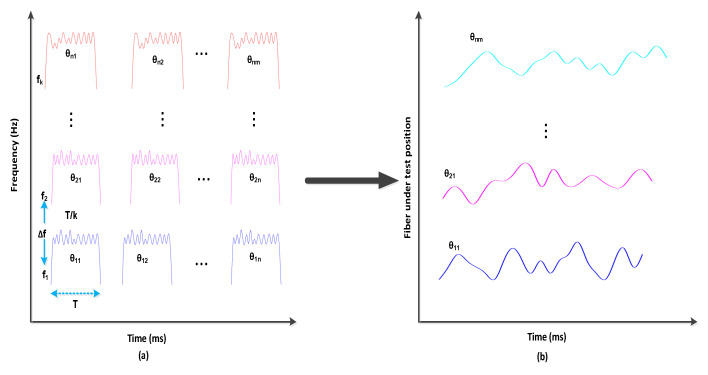
(**a**) Trace OTDR wave processing. (**b**) After STFT.

**Figure 8:**
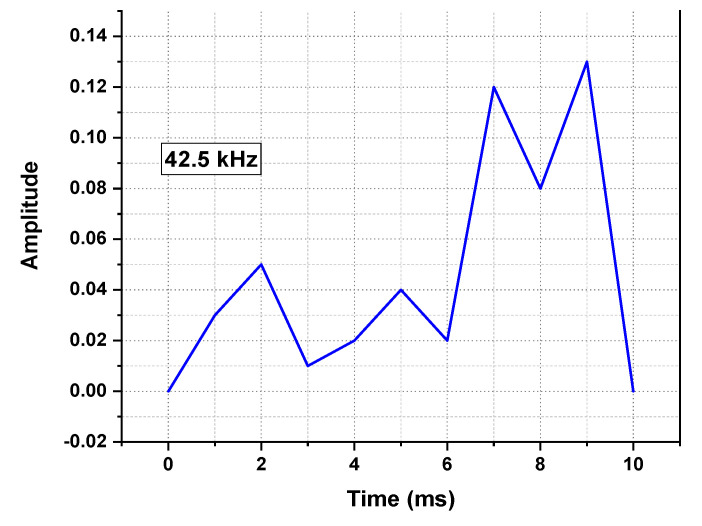
Amplitude vibration traces at different instants in time and 42.5 kHz frequency.

**Figure 9:**
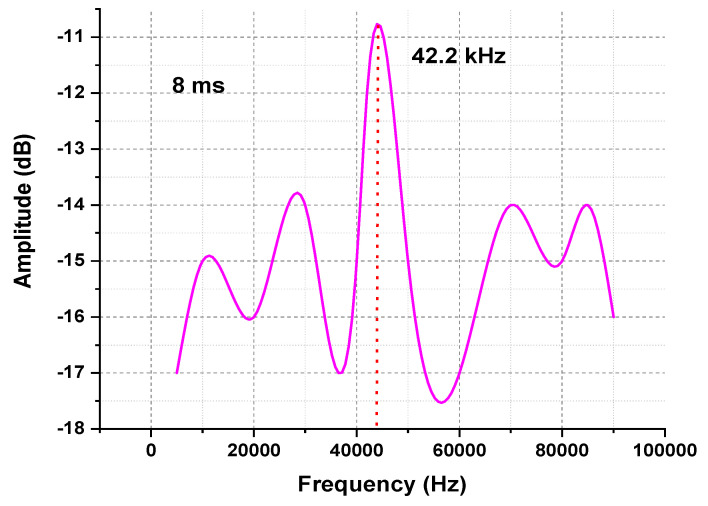
Amplitude vibration traces at different instant frequencies and 8 ms time.

**Figure 10:**
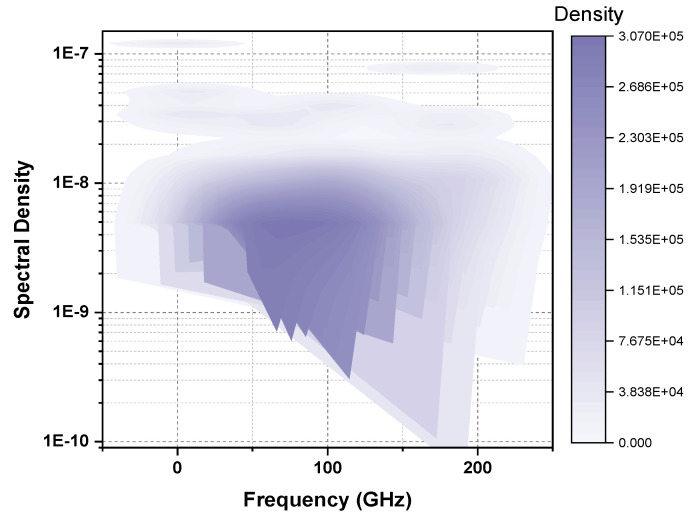
Estimation of vibration spectral density.

**Figure 11:**
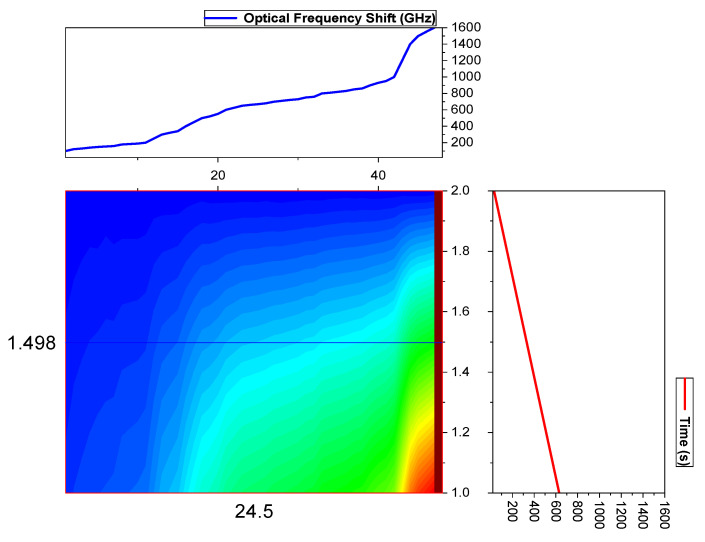
Experimental analysis of the implemented optical fiber for communication.

The configuration of OTDR-coherent FDM vibration sensing is declared in Figure 5 for long path estimations. The narrow band input light is divided into two parts using an optical coupler, where one part of the light is used for local light to detect heterodyne while the second division of light is used as a test light. To get the coherence length of light, the beat signal phase is kept constant, and the phase of the backscattered light is maintained. The single-sideband modulator is used for coding the test light with the help of frequency multiplexing. This procedure provides flexibility in experimental and vibration computations and shows a clear difference from the mechanisms discussed in [16,24,25,26]. As compared to the previous investigations, where electro-optical and acousto-optic modulators are used for frequency multiplexing and pulse inductions, respectively, this model produces the pulses by injecting a dummy frequency element among the multiplexed frequencies. Therefore, the essential procedures for vibration measurements can be achieved by a single modulator. The sequence of test pulse is mentioned in Figure 6, where several frequency parameters with *w* duration and frequency interval are organized in series with the same intervals. The $f_{0}$ dummy frequency is used to fill the pulse sequence, which has a different magnitude from the modulation frequencies $f_{i}$ to $f_{k}$. To make the space among the frequency component of the pulse sequence, the pulses are continuously inserted into the FUT at T/k intervals. The total duration *T* is kept greater than the round trip; to ensure that the same frequency element is not present in the vibration frequencies, the maximum number of multiplexed frequencies are required. By reason of the receiver bandwidth, the number of multiplexed frequencies are limited and can be measured as k/w. The beat signal current is realized by a photo-detector where the various frequency components are then divided into particular OTDR traces. The outcomes of the photo-detector are subjected to a short-time Fourier transform (STFT) to determine the complex phase of every frequency element. In the result of this applied procedure, the n×m OTDR traces are achieved, which are described in Figure 7. The applied signal processing to n×m OTDR tracer and the permutated n×m OTDR tracer are depicted in Figure 7. The amplitude vibration trace is presented at different instants in time in Figure 8. For this reason, the 42.5 kHz frequency was selected, which declared that the maximum variations occurred among 8 and 9 ms. Figure 9 shows the analysis of amplitude vibrations in dB at various frequencies in Hz and 8 ms time. Here, the maximum change in amplitude is recorded at 42.2 kHz. The spectral density estimations for optical frequency are depicted in Figure 10, and this figure clarifies that high fluctuations in frequency enhance the spectral density. The experimental analysis of the proposed framework on the implemented optical fiber is shown in Figure 11 using time and distance for the evaluation of the optical frequency phase shift. The optical spectral structure is measured, which shows that the optical frequency shift is performed accurately even with a slight change in polarization because of external pressure. The measurement results are analyzed for both aerial and underground optical fiber cables implemented for communication.

## 3. Conclusions

Optical fiber communication has resolved long-distance transmission problems. However, when applying the optical cables for long-range transmission, the optical fiber may be disturbed by external physical loads. DVS technologies based on OTDR and OFDR techniques are suggested in this paper to minimize the dispersion and scattering losses due to external physical pressure. Different vibration states are applied to detect the failure inside the optical fiber and its position. Averaging the waveform to improve the vibration measurement is studied based on the frequency-multiplexed OTDR. The drawbacks of OFDR, like the low repetition rate of the probe light and short measurement range, are addressed by applying single-sideband modulation using a microwave signal generator. Relative distance estimations improve the distance measurements to a delay fiber.

## Data Availability

The data can be provided as per request.
